# Supplementary material for: Alterations in promoter interaction landscape and transcriptional network underlying metabolic adaptation to diet
Source: Nat Commun. 2020 Feb 19;11:962. doi: 10.1038/s41467-020-14796-x (PMC7031266; doi:10.1038/s41467-020-14796-x)
Supplement: Supplementary file 2 — Supplementary Information [file 41467_2020_14796_MOESM2_ESM.docx]

Supplementary Figures for “Alterations in promoter interaction landscape and transcriptional network underlying metabolic adaptation to diet” by Qin et al.

Supplementary Figures

**
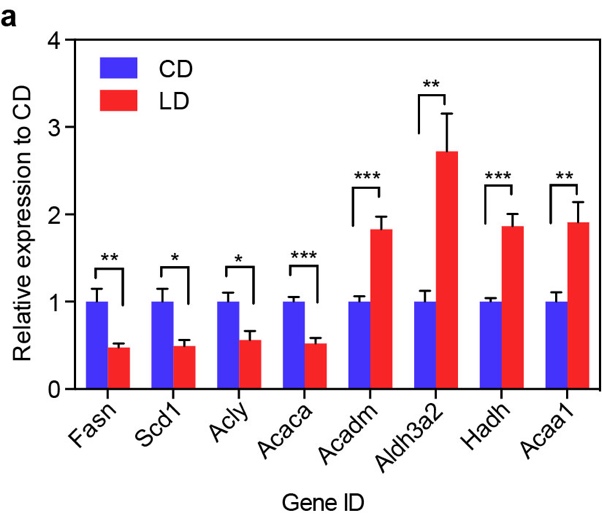
**

**Supplementary Figure 1 Real time PCR validation of differentially expressed genes**.

**a** Differential expressed genes identified in carbohydrate-rich diet (CD) or lipid-rich diet (LD) group. The column graph depicts mean and SEM (n=5 animals per group) with significance (2-tailed t test) indicated. The value in the carbohydrate-rich diet was set to one. ∗ p < 0.05, ∗∗ p < 0.01, ∗∗∗ p < 0.001

**
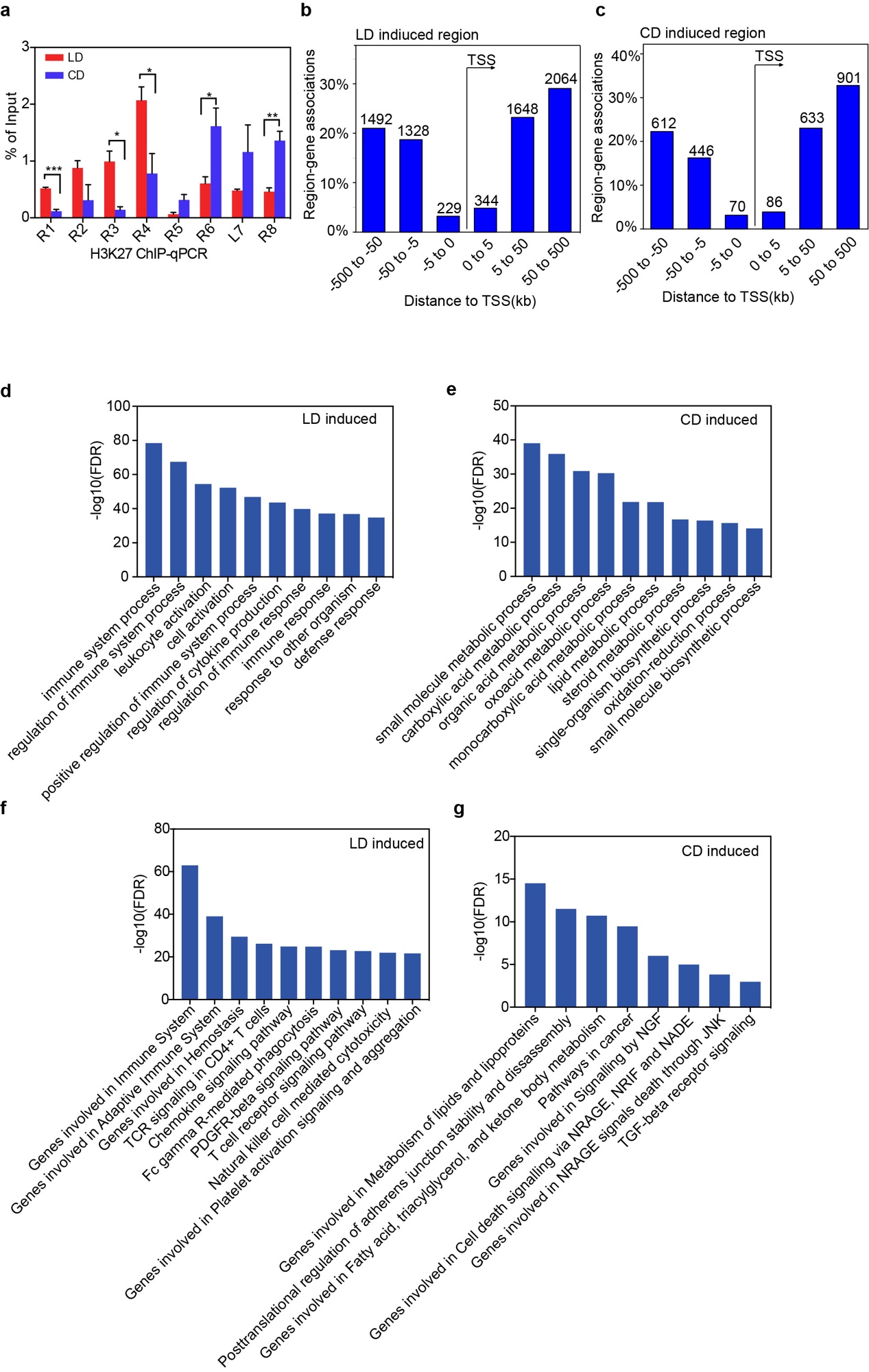
**

**Supplementary Figure 2 GO and MSigDB analysis of H3K27ac differentially enriched regions.**

**a** ChIP-qPCR validation of top eight diet induced H3K27ac differential bound regions. ChIP enrichment is expressed as percent input (n=3 animals per group). **b, c** Localization of diet-induced H3K27ac differentially enriched regions relative to gene transcription start sites. **d, e** GO analysis of of diet-induced H3K27ac differentially enriched regions. **f, g** MSigDB analysis of diet-induced H3K27ac differentially enriched regions.

**
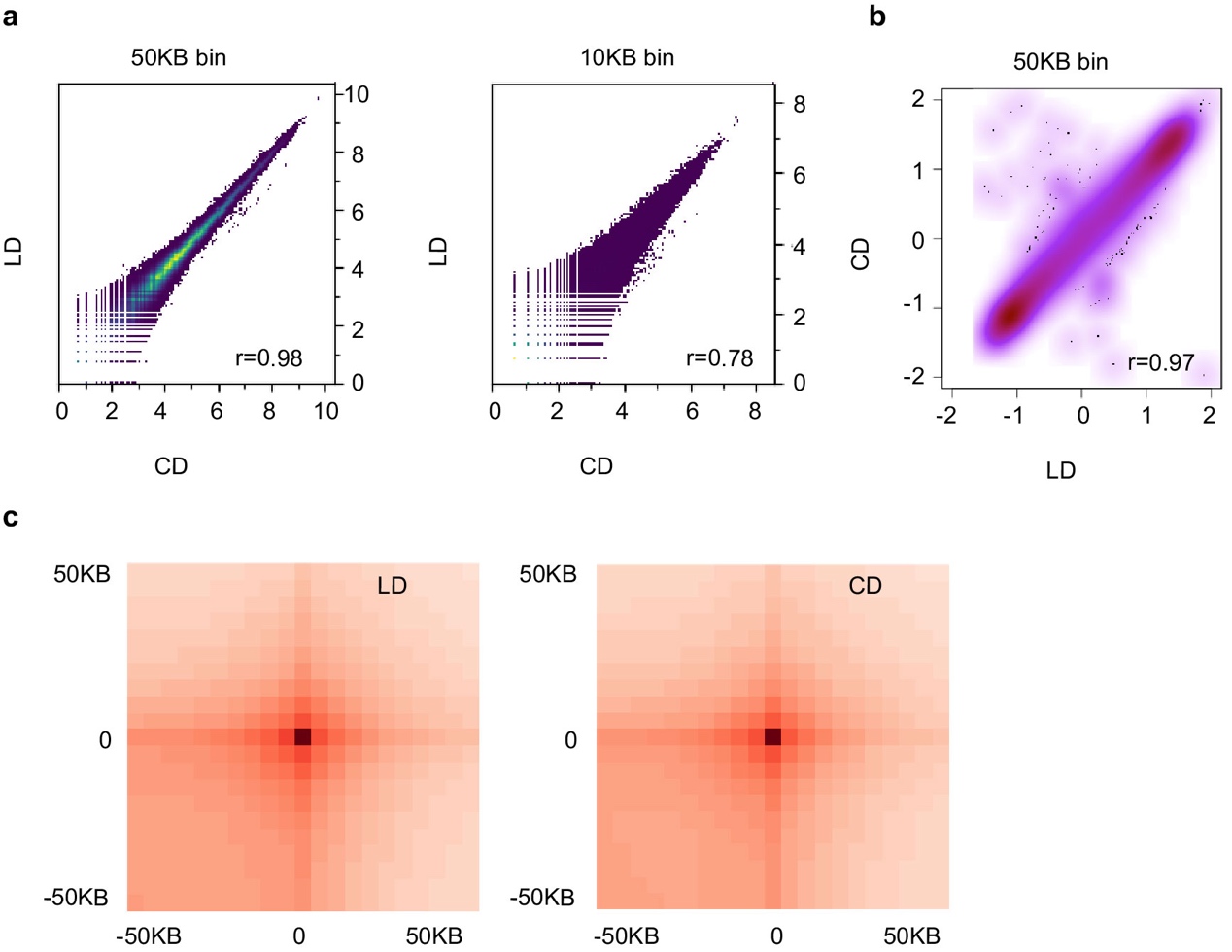
**

**Supplementary Figure 3 High order chromatin organization in liver.**

**a** The correlation of contact matrix between carbohydrate-rich diet (CD) or lipid-rich diet (LD) in the 50 kb bin and 10 kb bins. **b** Compartment A/B analysis in CD or LD group. **c** Aggregate peak plots of significant chromatin interactions in CD and LD group.


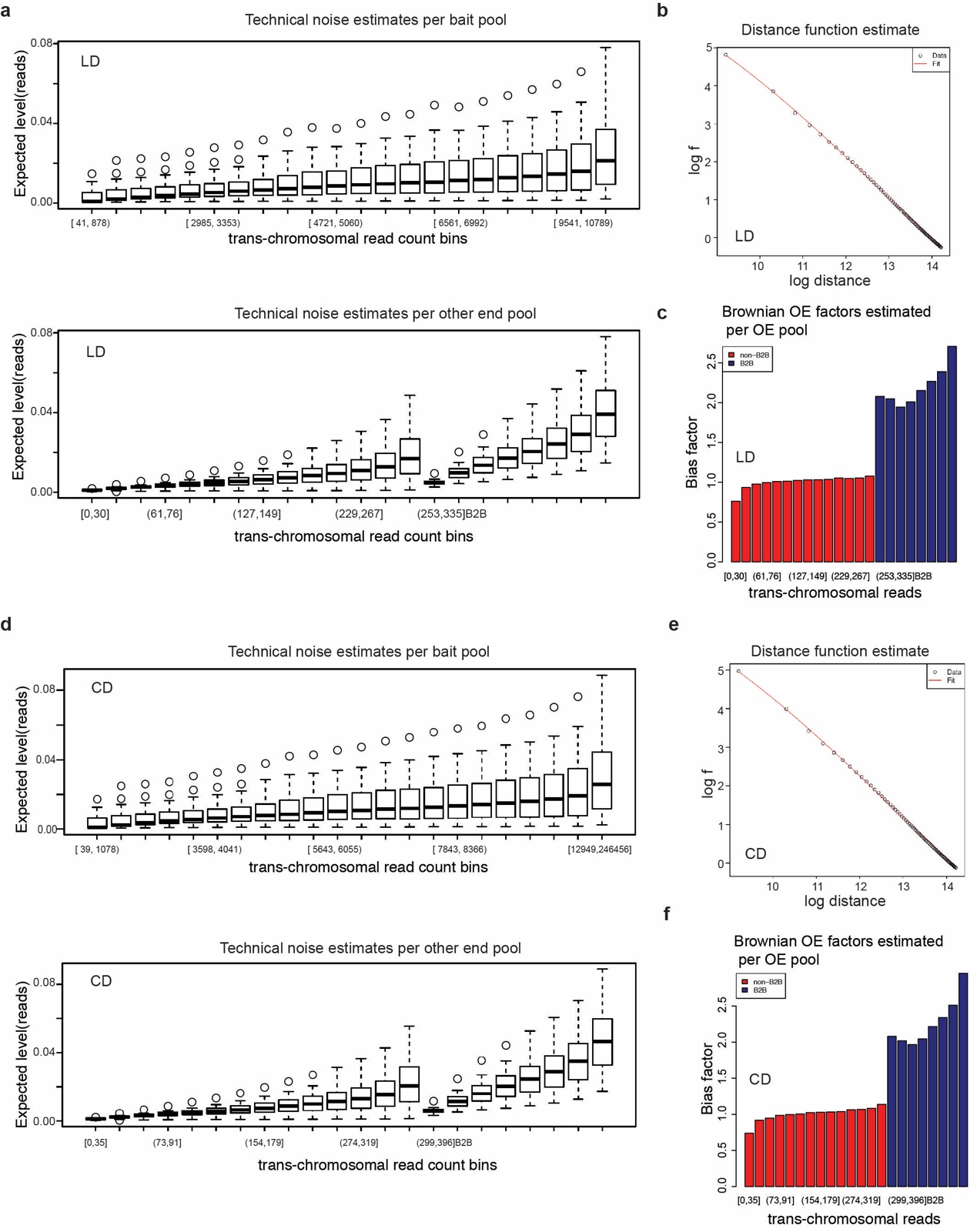


**Supplementary Figure 4 Capture Hi-C data quality accessed by CHiCAGO.**

**a, d** Technical noise in LD and CD was estimated separately for each combination of bait and other-end pools, each of noise was defined by the number of accumulated trans-chromosomal read pairs. The level of technical noise was a small fraction in both groups. **b, e** Mean number of Brownian reads expected for an average bait in LD and CD. **c, f** Multiplicative other-end bias (each bar represents a pool of other ends defined by the numbers of trans-chromosomal read pairs accumulated by each other end), the adjustment made to the mean Brownian read count, estimated in the pools of other ends.


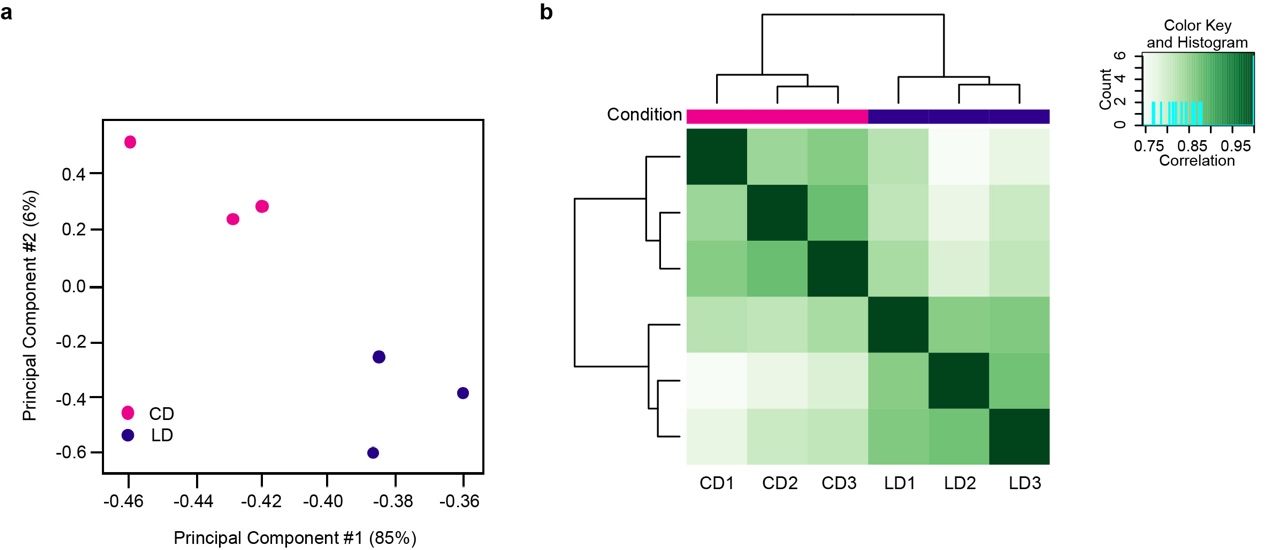


**Supplementary Figure 5 The PCA analysis and correlation analysis use the H3K27ac ChIP-seq data.**

**a** PCA plot using affinity data for all H3K27ac binding peaks. **b** Correlation heatmap, using all H3K27ac binding peaks.
