# Supplementary material for: Alterations in promoter interaction landscape and transcriptional network underlying metabolic adaptation to diet
Source: Nat Commun. 2020 Feb 19;11:962. doi: 10.1038/s41467-020-14796-x (PMC7031266; doi:10.1038/s41467-020-14796-x)
Supplement: Supplementary file 3 — Description of Additional Supplementary Files [file 41467_2020_14796_MOESM3_ESM.pdf]

Description of Additional Supplementary Files

File Name: Supplementary Data 1

Description: Differentilal expressed genes (LD vs CD) from DEseq2.

File Name: Supplementary Data 2

Description: a - Overlap of H3K27ac peaks between each replicate

b - H3K27ac differetilally enriched regions(LD vs CD) from Diffbind

File Name: Supplementary Data 3

Description: a - Metrics of Hi-C and CHi-C sequencing and processing by HiCUP.

b - Genomic coordinates of topological domains(TAD) mapped in LD and CDHi-C libraries.

c - Rewied promoter interactions by Diet

File Name: Supplementary Data 4

Description: a - Hnf4a differetilally enriched regions (LD vs CD) from Diffbind

b C/EBPa differetilally enriched regions (LD vs CD) from Diffbind

File Name: Supplementary Data 5

Description: a - Primers for qPCR

b - Primers for ChIP-qPCR

File Name: Supplementary Data 6

Description: Metrics of ChIP-seq data in current study

File Name: Supplementary Data 7

Description: The caloric breakdown of the three diets employed in this study are presented along with details of the formulation for each specific regimen.
